# Sojourn-time-corrected receiver operating characteristic curve (ROC) for prostate specific antigen (PSA) test in population-based prostate cancer screening

**DOI:** 10.1038/s41598-020-77668-w

**Published:** 2020-11-26

**Authors:** Hsiao-Hsuan Jen, Wei-Jung Chang, Chen-Yang Hsu, Amy Ming-Fang Yen, Anssi Auvinen, Tony Hsiu-Hsi Chen, Sam Li-Sheng Chen

**Affiliations:** 1 Graduate Institute of Epidemiology and Preventive Medicine, College of Public Health, National Taiwan University, Taipei, Taiwan; 2 College of Nursing, National Taipei University of Nursing and Health Science, Taipei, Taiwan; 3 School of Oral Hygiene, College of Oral Medicine, Taipei Medical University, Taipei, Taiwan; 4 Unit of Health Sciences, Faculty of Social Sciences, Tampere University, Tampere, Finland; 5 TMU Research Center of Cancer Translational Medicine, Taipei Medical University, Taipei, Taiwan

**Keywords:** Cancer screening, Predictive markers

## Abstract

Evaluating the performance of serum prostate-specific antigen (PSA) test in population-based screening with receiver operating characteristics (ROC) curve often neglects the time dimension. Asymptomatic cases with negative PSA test would have been missed if sojourn time is not taken into account to allow for cases surfacing into the clinical phase. Data included 20,796 men with PSA test at the first screening round was used from population-based Finnish prostate cancer screening trial during 1996–1999. Cancers detected at the first screen, together with interval cancers ascertained during 4-year follow-up were expediently used to estimate sensitivity and specificity. A sojourn-time-corrected model was applied to estimating the possible false negative cases for those with PSA < 4 ng/ml for correcting the ROC curve. The estimated sensitivity estimate was reduced from 94.4% without correction to 68.8% with correction but the estimated specificity was identical (89.4% vs. 89.2%) at cutoff of 3 ng/ml. The corrected area under curve (AUC) [77.0% (74.9–79.1%)] of the PSA test was significantly lower than the uncorrected AUC [95.9% (95.3–96.6%)]. The failure of considering the time since last negative screen due to incomplete ascertainment for asymptomatic cancer led to the overestimation of PSA test performance that further affects the cut-off value of PSA tests for population-based prostate cancer screening.

## Introduction

Incomplete ascertainment of prostate cancer (PrCa) using the prostate-specific antigen (PSA) test for clinical patients was noted^1^. The sensitivity of the PSA test at the selected threshold may be overestimated^1^ if fewer men with negative results undergo confirmatory diagnosis with biopsy because false negative cases may be incompletely ascertained. This incomplete ascertainment renders the receiver operating characteristic (ROC) curve inaccurate for evaluations of sensitivity, specificity, and area under curve (AUC). To correct this ascertainment bias, two studies performed biopsies for the entire range of PSA results to ascertain PrCa below the cutoff of the PSA test^2,3^. However, the application of PSA to population-based PrCa screening to provide confirmatory diagnosis with biopsy to identify false negative cases from a large proportion of test negative cases (PSA below cutoff level) is not feasible due to the invasiveness of biopsy and the enormous costs involved. Incomplete ascertainment results in a lower sensitivity, and up to 25% of men with PSA < 4 harbored PrCa in the Prostate Cancer Prevention Trial^4^. The results of a conventional analysis of test performance using the ROC curve would be also affected if these undetected and missed cancers are not included in the analysis. Therefore, the performance of a PSA test using a pre-determined cutoff as a population-based screening test would be overestimated without correction of this incomplete ascertainment bias.

One solution is to ascertain cancers arising from false negative cases via the linkage of the test-negative cohort with a cancer registry. A previous method using a fixed follow-up time was proposed to cope with incomplete ascertainment bias^1^. However, this approach is not theoretically sound because the follow-up time for allowing these missed cancers to surface as clinical cases is dependent on the length of the follow-up time required for a valid estimation of risk after a negative screening test, which is subject to the rate of disease progression from a pre-clinical detectable phase (PCDP, cancer in an asymptomatic phase that may be detected by a test) to the clinical phase (CP, cancer in a symptomatic phase), namely mean sojourn time (MST), which is the average duration of staying in PCDP^5,6^. The estimates of MST for PrCa is rather long at approximately 6.4–6.75 years^6,7^. This duration means that false negative cases may be incompletely ascertained if the follow-up time is not sufficient compared based on the long MST for PrCa. Consideration of MST in combination with the pre-clinical incidence rate using PSA level allows for the correction of sensitivity, specificity, and the ROC curve of PSA tests even in cases of incomplete ascertainment of cancers below a pre-determined cut-off for PSA (screen-negative men). The use of a multi-state model would consider PSA test information and incorporate genetic information to develop personalized screening for PrCa^8^.

Therefore, we applied a new quantitative sojourn time-based method to ascertain the number of missing asymptomatic cancer cases that arise in subjects with a negative PSA result after screening to correct the sensitivity and specificity of this test and the resulting ROC curve.

## Results

### Cancer found by PSA level

A total of 584 PrCa cases were detected by screening with the additional 129 interval cancers ascertained between the 1st and 2nd screenings (Table 1). These 713 cases were used in the estimation of sensitivity and ROC curve analysis. Of the 713 PrCa cases, 40 interval cancers occurred in the 17,890 men with PSA < 3 ng/ml, with an incidence rate of 2.2 per 1000 man-years, which is dramatically lower than 240.6 per 1000 based on the finding of 584 screen-detected and 89 interval cancers in 2797 men with PSA ≥ 3 ng/ml.

**Table 1:** Cancer detected and found by PSA level at first round of Finnish prostate cancer screening trial.

PSA (ng/ml)	First round		
	Participants	SD	IC
0.0–2.9	17,890	–	40
3.0–3.9	1064	36	22
4.0	1733	548	67
Total	20,687	584	129

SD: number of screen-detected cancers, IC: number of interval cancers.

### Uncorrected receiver operativing characteristic curve of PSA

**Figure 1:**
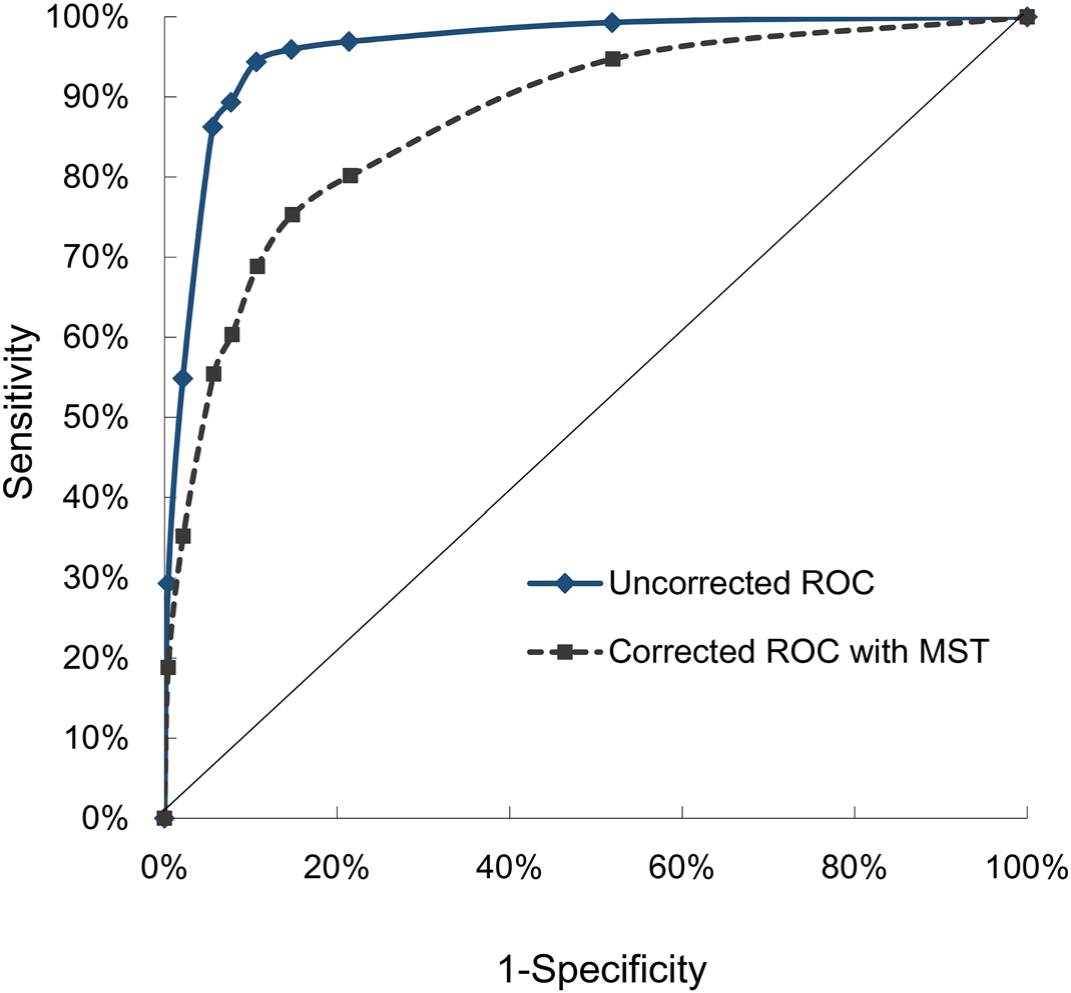
Uncorrected and corrected ROC curves for population-based PSA screening.

In the naïve estimation of the diagnostic performance based on the above figures and ignoring MST, the sensitivity for a cut-off of 4 ng/ml was 86.3%, and specificity was 94.4%. For a cut-off of 3 ng/ml, the corresponding figures were sensitivity of 94.4% and specificity of 89.4% (Table 2). Figure 1 shows that the AUC of the PSA test was 95.9% (95% confidence interval [CI]: [95.3%, 96.6%]). The optimal cut-off based on the uncorrected ROC curve was 3 ng/ml.

**Table 2:** Uncorrected sensitivity, specificity, PPV and NPV for prostate cancer by different cut-off PSA.

PSA ≥ (ng/ml)	Uncorrected				
	Sen (%)	Spe (%)	PPV (%)	NPV (%)	Youden index (%)
1.0	99.3	48.1	6.4	99.9	47.4
2.0	96.9	78.6	13.9	99.9	75.5
2.5	95.9	85.3	18.9	99.8	81.2
3.0	94.4	89.4	24.1	99.8	83.8
3.5	89.3	92.3	29.3	99.6	81.6
4.0	86.3	94.4	35.5	99.5	80.7
6.0	54.8	97.9	48.0	98.4	52.7
10.0	29.3	99.6	71.6	97.5	28.9

Sen: sensitivity; Spe: specificity; PPV: positive predictive value; NPV: negative predictive value; Youden index: Sensitivity + Specificity − 1.

### Estimated expected PCDP cancers and corrected receiver operating characteristic curve of PSA

To correct the diagnostic performance, 18,954 men with PSA < 4 ng/ml were divided into six groups according to PSA level. The higher the PSA level, the higher likelihood of PCDP cancers between the two screening rounds were observed. Table 3 shows the corresponding estimate of incidence of PCDP ($\lambda_{1}$) and expected PCDP cancers. Given 6.75 years of MST, the overall expected PCDP cancers are 417 cases. The sensitivity analyses showed that more PCDP cancers are expected with longer MST.

**Table 3:** Estimated pre-clinical detectable phase cancers by PSA level.

PSA (ng/ml)	Number	Observed ICs	Incidence of PCDP ($\lambda_{1}$)	P_PCDP_	Estimated PCDP cases
0.00–0.99	9614	5	0.000528	0.0036	34
1.00–1.99	6112	17	0.002779	0.0188	115
2.00–2.49	1342	7	0.005128	0.0346	46
2.50–2.99	822	11	0.012479	0.0842	69
3.00–3.49	623	13	0.018583	0.1254	78
3.50–3.99	441	9	0.018225	0.1230	54
Total	18,954	62	0.003258	0.0220	417

ICs: Interval cancers, P_PCDP_: Probability of cancer stayed at detectable phase, MST: Mean sojourn time.

After the expected detectable cancers that arose in subjects with PSA < 4 ng/ml were taken into account, the sensitivity and specificity for the PSA cutoff at 4 ng/ml were reduced to 55.4% and 94.3%, respectively (Table 4). The corresponding figures were 68.8% and 89.2%, respectively, with the cutoff at 3 ng/ml. The AUC for the corrected ROC curve was 77.0% (95% CI: [74.9%, 79.1%]). The corrected optimal cut-off based on the ROC curve was 2.5 ng/ml, where the sensitivity was 75.3% and specificity was 85.2% (Fig. 1). The difference in AUC between the uncorrected and corrected estimates was 18.9% (95% CI: [16.7%, 21.1%], *p* < 0.001), with the corrected AUC being significantly smaller than the uncorrected AUC. The sensitivity analyses for varying MST found that the difference between uncorrected and corrected AUCs varied from 15.0% (95% CI: [12.9%, 17.2%]) with 5-year MST and 19.4% (95% CI: [17.2%, 21.7%]) with 10-year MST. The detailed number of expected detectable cancers and the corresponding ROC curves giving different estimates of MST are shown in Supplemental Table 1 and Supplemental Fig. 1.

**Table 4:** Corrected sensitivity, specificity, PPV and NPV for prostate cancer by different cut-off PSA.

PSA ≥ (ng/ml)	Corrected				
	Sen (%)	Spe (%)	PPV (%)	NPV (%)	Youden index (%)
1.0	94.7	48.0	6.4	99.6	42.8
2.0	80.2	78.5	13.9	98.9	58.6
2.5	75.3	85.2	18.9	98.7	60.5
3.0	68.8	89.2	24.1	98.3	58.1
3.5	60.3	92.2	29.3	97.7	52.5
4.0	55.4	94.3	35.5	97.4	49.7
6.0	35.2	97.8	48.0	96.4	33.1
10.0	18.8	99.6	71.6	95.6	18.4

Sen: sensitivity; Spe: specificity; PPV: positive predictive value; NPV: negative predictive value; Youden index: Sensitivity + Specificity − 1.

## Discussion

Our results corrected the performance of the PSA test by considering the time dimension of the occurrence of interval cancer from time since the last negative screen due to incomplete ascertainment of asymptomatic cases staying in the pre-clinical phase. We used population-based screening data and interval cancer without correcting this bias and revealed that the sensitivity (94.4% and 86.3% for PSA ≥ 3 and 4 ng/ml, respectively) and AUC (95.9%) of PSA tests were overestimated. The very higher AUC would mislead us about the good performance of PSA test. After correction, the sensitivity and AUC were reduced to 68.8%, 55.4%, and 77.0%, respectively. Our corrected estimates may explain the very high estimates of the AUC for PSA tests in two population-based case–control studies^9,10^ that did not consider this type of bias. The main interest of the present study was to correct the diagnostic performance of the PSA test in the setting of screening. Although different method such as using relative statistics can also apply to estimate the corrected sensitivity by comparing screening tests^11^, our method was developed to correct the diagnostic performance of a biomarker considering time dimension by only using an interval scale between first and second screen for simplification. We used the observed interval cancers to estimate missing cases, which came from patients in a symptomatic clinical phase but not in PCDP and was not related to other verification methods^1^. This correction for the time dimension is also important when considering interval cancers using the verification method for adjusting for differential or non-differential misclassification. It is unreasonable to assume that interval cancers during a 4-year screening interval covered all false negative cases, such as PrCa with a long MST. The diagnostic performance was estimated using our method, which is suitable for national-wide population-based screening when disease prevalence and natural history are fixed. It is more practical for application in public health. The present study corrected for the incomplete ascertainment of false negative cases in population-based screening compared to most previous studies that used the verification of bias adjustment method based on a clinical series patients with or without disease-free control^1–3,12^.

In contrast to previous studies were often limited to subjects with PSA ≥ 4 ng/ml^1,13–15^, our study included PSA values below 4 ng/ml. However, if we considered patients with PSA below 4 ng/ml, the evaluation of the performance of PSA tests even with correction for verification bias (selective referral to biopsy of only screen-positive men) also must consider the time dimension since the last negative screen for interval cancer to avoid bias. For example, a study in the U.S. followed healthy men with initial PSA < 3 ng/ml in the placebo group of a randomized control trial (for prostate prevention using medication) and provided biopsy in 7 years. The low sensitivity of 20.5% for the subsequent PSA ≥ 4.1 ng/ml was likely attributed to the exclusion of men with a high initial PSA from the study^4^ and the failure to consider time since the last negative screening for interval cancers in non-biopsied men as disease-free.

Some studies used correction for verification bias due to biopsy being limited to men with high PSA using a differential misclassification method. Punglia et al. reported AUCs of 62% and 69% for men aged over and under 60 years old, respectively, based on biopsied men and men with interval cancers during 18 months of follow-up^1^. The AUCs increased to 72% and 86% for elderly and younger men after correcting for verification bias using a misclassification method. These estimates may still be biased because not all interval cancers were observed due to incomplete ascertainment of asymptomatic cancer in the previous screen. This omission may explain the slightly lower performance estimates for PSA tests compared to the AUC of 77.0% in the present study.

Correction for this incomplete ascertainment bias also affects the selection of the optimal PSA cut-off based on ROC analysis. The optimal PSA cutoff in the uncorrected analysis was estimated as 3 ng/ml, and it was reduced to 2.5 ng/ml after correction because only very few interval cancers were observed for a PSA below this level. Nevertheless, the selection of cut-off cannot entirely rely on the optimal point identified from the ROC curve because it gives a similar weight to false-positive and false-negative results, which may be misleading. This result also suggests that the threshold of 4 ng/ml, which is widely used in clinical decision-making, may not be adequate for disease detection^10,15,16^.

Our sojourn-time-corrected ROC method was essentially based on the three-state disease progressive model. The three-state disease progressive model is widely used in breast cancer screening^17–21^. The homogeneous Markov model with two exponential distributed time durations was developed, and a general formula that allows for a time-heterogeneous property was derived. Our method would have further benefited from the use of general formulas when informative data supports its use. On the other hand, our method was developed to correct the diagnostic performance of a biomarker using an interval scale in the screening that uses first-round screen data only, but our purpose was not to evaluate the schedule sensitivity of a periodic screening program. Notably, screening is not a one-time event, and other methods that take more information from repeated screens may be applied to answer the schedule sensitivity of periodic screening^22^.

There are some limitations in our study. First, our method was based on the assumption that MST is known. The generalizability of the findings may be limited by the MST of 6.75 years, which was estimated from a previous study of the Finnish trial^6^ and may not be applicable to other ethnic groups. The results of sensitivity analyses showed that longer MSTs result in further separation of the corrected ROC from the uncorrected one. Second, MST may be dependent on PSA level. However, due to sparse information, we assumed that MST was independent of PSA level. Nonetheless, our proposed method may be further generalized to the scenario of PSA-dependent PSA. Third, the proposed model must be validated via external validation for generalization. A sampling designed population-based screening with the use of synchronous tests and confirmatory exams is further needed.

In conclusion, we demonstrated that AUC decreased after correction for detectable cancers when biopsy was performed on all screen-negative men. Sensitivity and AUC were also lower after this correction. These novel methods may help policy-makers determine the cut-off value of PSA tests for population-based PrCa screening.

## Materials and methods

### Data sources

Data were derived from the Finnish PrCa screening trial, which is the largest component of the European Randomized Study of Prostate Cancer screening^23^. It began in 1996 in two metropolitan regions, Helsinki and Tampere, in Finland. A total of 80,458 men born in 1929–1944 and identified from the Population Register Centre of Finland comprised the study population. In the beginning of each calendar year (1996–1999), men aged 55, 59, 63 or 67 years were randomly allocated to the screening and the control arms. There were 32,000 men in the screening arm and 48,458 in the control arm. Invitations to participate in the study were mailed to the men at a four-year interval in the screening arm. There were 20,796 men who participated in the first screening round. After informed consent was obtained, a blood sample was drawn at the local clinic. Serum PSA concentrations were analyzed at the Central Laboratory of Helsinki University Hospital using Tandem-E (Beckman-Hybritech, San Diego, CA, USA) assays for the determination of total PSA and Delfia (Wallac, Turku, Finland) assays for free PSA, if indicated, as an ancillary test^24,25^.

### The retrospective cohort using the Markov model for estimating MST

**Figure 2:**
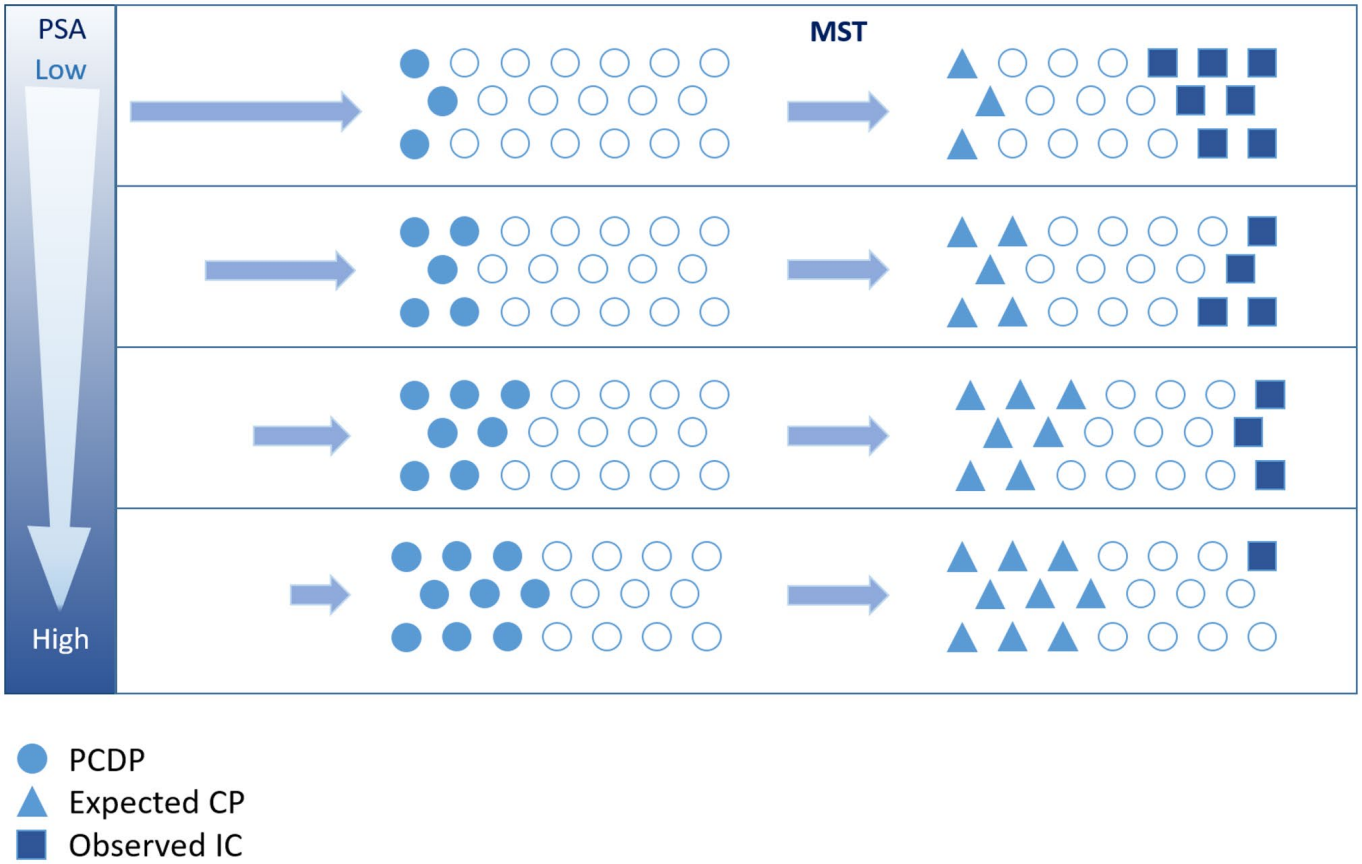
Age-PSA-dependent and sojourn-time-corrected design.

Figure 2 shows our use of the retrospective cohort for estimation of the pre-clinical incidence rate of PrCa and the MST to estimate the number of cases missed in screening at the pre-clinical detectable phase. In this retrospective cohort, men with serum PSA ≥ 4 ng/ml were referred to hospitals for all three diagnostic examinations: digital rectal examination (DRE), trans-rectal ultrasound (TRUS) and trans-rectal prostate biopsy. Men with serum PSA concentrations 3.0–3.9 ng/ml were referred for ancillary testing, which included DRE during the first 3 years of the study (1996–1998) and free-to-total PSA ratio beginning in 1999 with a cutoff of 16%. Men with a positive ancillary test were also referred for diagnostic examinations.

To cope with the incomplete ascertainment of PrCa below the pre-determined cut-off of the PSA test in population-based PrCa screening, the PSA-negative cohort requires a follow-up time. The false negative cases are dependent on the length of follow-up time and associated with MST, which may also be expressed as the average duration that the cancer stays in the preclinical detectable phase^5–7^. The cancers detected at screening, which are generally called screen-detected cancer, were occult cancers in the preclinical detectable phase. The false negative cases in population-based cancer screening may be defined as interval cancer, which was missed at screening or progressing to symptomatic cancer based on the follow-up time. Therefore, false negative cases may be incompletely ascertained when the follow-up time is not considered on the basis of MST.

The current study defined interval cancers as subjects who had negative PSA results at the first screen but developed symptoms and confirmed PrCa before their second scheduled screen. Notably, some cases with positive PSA results at the first screen and confirmed PrCa more than 1 year after the first screen were also defined as interval cancers. They were identified via record linkage with the Finnish Cancer Registry, which is a nationwide, population-based cancer registry with practically complete coverage of cancer cases, especially solid tumors, in Finland^26^. The ROC for population-based PSA screening was uncorrected based on the fixed follow-up time mentioned above.

Because the long sojourn time is a unique characteristic of PrCa, cancer should be detected by estimating the incidence of cancer at a pre-clinical detection phase to allow for MST adjustment beyond confirmatory diagnosis with biopsy. Considering the MST in combination with the pre-clinical incidence rate using the PSA level allows for corrections of sensitivity, specificity, and the ROC curve of the PSA test. The multi-state Markov model estimated that the incidence rate of the PCDP PrCa using PSA level below the cutoff was estimated to ascertain the under-detected cancers in the screening of negative cases. The details of the modelling methodology are elucidated in the Supplemental material. Therefore, the corrected ROC is plotted after the correction of sensitivity.

The present study used the data of men who participated in the first screen, PrCa cases identified in the first screen and interval cancers that occurred before the second scheduled screen. We excluded 102 men with positive screen results that were not confirmed by biopsy procedure and 7 men with a PSA < 3 ng/ml and biopsy as a result of positive DRE results at the beginning of study. The remaining 20,687 subjects were used in further analysis.

### Statistical analysis

Results from the first round screening were used for evaluating the sensitivity, specificity, and the ROC curve of the serum PSA test. Men with screen-detected cancer or interval cancer during the subsequent 4 years were considered subjects with disease. Others without interval cancers, including men with negative biopsy results and men without biopsy because of a negative screen test, were all considered disease-free. Naïve sensitivity was calculated as the screen-detected cancers divided by subjects with PrCa (= screen-detected cancers + interval cancers). The naïve specificity was calculated with the numerator as true negative cases, and disease-free subjects as the denominator. The naïve ROC curve was plotted as 1—specificity versus sensitivity for all cutoff values in the range of PSA levels by every 0.1 ng/ml difference.

The under-detected cancers in the screening of negative cases were obtained via estimation of the incidence rate of the PCDP PrCa using Eq. (5) in the supplementary information. Equation (5) shows that the incidence rate of PCDP PrCa was a function of the progression of PCDP to CP PrCa given the observed interval cancer rate. This function in Eq. (5) was derived from Eqs. (1)–(4). Based on Eqs. (6)–(7), the expected number of asymptomatic PrCa for screened men with a PSA level below 4 ng/ml at the first screen was estimated using Eq. (8), and the corrected sensitivity and specificity were obtained as follows:

$$ \begin{aligned} \overline{SEN}_{i} &= \frac{{N_{i}^{TP} }}{{N_{i}^{TP} + N_{i}^{FN} + N_{{PCDP_{i} }}^{{PSA_{1} < 4}} }} \hfill \\ \overline{SPEC}_{i}& = \frac{{N_{i}^{TN} - N_{{PCDP_{i} }}^{{PSA_{1} < 4}} }}{{N_{i}^{FP} + N_{i}^{TN} - N_{{PCDP_{i} }}^{{PSA_{1} < 4}} }} \hfill \\ \end{aligned} $$

where

$$\overline{SEN}_{i}$$:
Updated Sensitivity at *i* cut-off PSA level.
$$\overline{SPEC}_{i}$$:
Updated Specificity at *i* cut-off PSA level.
$$N_{i}^{TP}$$:
Numbers of true positive cases before correction at *i* cut-off PSA level.
$$N_{i}^{TN}$$:
Numbers of true negative cases before correction at *i* cut-off PSA level.
$$N_{i}^{FP}$$:
Numbers of false positive cases before correction at *i* cut-off PSA level.
$${\text{N}}_{{{\text{PCDP}}}}^{{{\text{PSA}}_{{1}} < 4}}$$:
Expected number of asymptomatic PrCa for the screened men with PSA levels below 4 ng/ml.

To better understand this calculation, we took men with PSA levels less than 1 ng/ml as an example. At prevalent screen (N = 9614), the probability of interval cancers during the four years of inter-screening interval (P_IC_(4)) was estimated as 0.00052 (= 5/9614). Given 6.75 years of MST and the probability of interval cancers (P_IC_(4) = 0.00052), the incidence rate of PCDP (0.000528) was obtained using Eq. (5). Based on the Eq. (7), the probability of cancer staying at PCDP for those under 60 years old was 0.0036. Therefore, the expected number of PCDP cases was estimated as the total number of different PSA levels multiplied by the probability of cancer staying at PCDP (9614 × 0.0036 = 34). For PSA levels less than 1 ng/ml, the 99.3% (708/713) sensitivity was corrected as 94.7% (708/(713 + 34)). The specificity changed from 48.1% to 48.0%. The details of the modelling methodology are elucidated in the Supplementary material.

The corrected sensitivity and specificity of PSA levels were calculated as described above. The corrected ROC curve was plotted according to a series of cutoffs. The area under the ROC curve, the corresponding 95% confidence intervals (CI), and the detection of differences between areas under two ROC curves were calculated using Hanley’s method^27^.

### Ethical consideration

We used data derived from a population-based screening for PrCa in Finland. The original research protocol was reviewed and approved by the ethical committees of Helsinki University Hospital and Tampere University Hospital. Written, informed consent was given by the participating men in the screening arm.

All methods were carried out in accordance with relevant guidelines and regulations.

## Supplementary information

Supplementary information.
