# Supplementary material for: Sojourn-time-corrected receiver operating characteristic curve (ROC) for prostate specific antigen (PSA) test in population-based prostate cancer screening
Source: Sci Rep. 2020 Nov 26;10:20665. doi: 10.1038/s41598-020-77668-w (PMC7691355; doi:10.1038/s41598-020-77668-w)
Supplement: Supplementary file 1 — Supplementary information. [file 41598_2020_77668_MOESM1_ESM.pdf]

Sojourn-time-corrected Receiver Operating Characteristic Curve (ROC) for Prostate Specific Antigen (PSA) Test in Population-based Prostate Cancer Screening

Hsiao-Hsuan Jen[1], Wei-Jung Chang[1], Chen-Yang Hsu[2,3], Amy Ming-Fang Yen[4], Anssi Auvinen[5], Tony Hsiu-Hsi Chen[1], Sam Li-Sheng Chen[4]

[1] Graduate Institute of Epidemiology and Preventive Medicine, College of Public Health, National Taiwan University, Taipei, Taiwan

[2] Master in Public Health, College of Public Health, National Taiwan University, Taipei, Taiwan

[3] College of Nursing National Taipei University of Nursing and Health Science

[3] School of Oral Hygiene, College of Oral Medicine, Taipei Medical University, Taipei, Taiwan

[5] Faculty of Social Sciences/Section of Health Sciences, University of Tampere, Tampere, Finland

Supplementary

Multi-state Markov model for estimation of cancer progression rate of test negative cases

The under-detected cancers arising from screen-negative cases that were re-captured by the use of the proposed three-state Markov model for the natural history of prostate cancer is depicted as follows:

$$\text{Free of Prostate Cancer (0)} \overset{\lambda_1}{\rightarrow} \text{PCDP (1)} \overset{\lambda_2}{\rightarrow} \text{CP (2)}$$

λ_1 and λ_2 represent annual transition rates of the incidence rate of the pre-clinical detectable phase (PCDP) prostate cancer (PrCa) and the progression from the PCDP to the clinical phase (CP) respectively. In this study, we assumed the transition rates are constant with time, which implies that the sojourn time follows exponential distribution. The inverse of λ_2 gives an estimation of mean sojourn time (MST)[1].

Using the stochastic model, we could estimate the under-detected cancer by estimation of the λ_1 and λ_2. Let X_i denote a random process of three-state model for the progression of PrCa with state space W={0,1,2}, 0: normal, 1: PCDP, 2: CP and i for detection modes denoted by

s_0: prior to 1st screen,

s_1: 1st screen,

s_{12}: between 1st and 2nd screens,

s_2: 2nd screen.

For example, X_{s12} =2 represents that the case were detected in the CP after 1st negative screen but before the second scheduled screen. For those with PSA<4ng/ml at 1st screen, the probability of being in PCDP at the 2nd screen or surfacing to CP before the scheduled 2nd screen can be expressed as follows.

$$\mathrm{P}((X_{s12} = 2 \text{ or } X_{s2} = 1) \cap (X_{s1} = 1 \text{ or } X_{s1} = 0) | X_{s0} \neq 2) \qquad (1)$$

It should be note that in the population-based screening program, we invited only those not being diagnosed as PrCa before their first screen. Therefore, the conditional probability of not in the CP prior to first screen ($X_{s0} \neq 2$) has to be obeyed.

Equation (1) can be further decomposed into the following formula,

$$\mathrm{P}\left((X_{s12} = 2 \cap (X_{s1} = 1 \text{ or } X_{s1} = 0))\middle| X_{s0} \neq 2\right)$$

$$+\mathrm{P}\left((X_{s2} = 1 \cap (X_{s1} = 1 \text{ or } X_{s1} = 0))\middle| X_{s0} \neq 2\right)$$

$$= \mathrm{P}\big((X_{s12} = 2 \cap X_{s1} = 1)\big| X_{s0} \neq 2\big) + \mathrm{P}\big((X_{s12} = 2 \cap X_{s1} = 0)\big| X_{s0} \neq 2\big)$$

$$+\mathrm{P}\big((X_{s2} = 1 \cap X_{s1} = 1)\big| X_{s0} \neq 2\big) + \mathrm{P}\big((X_{s2} = 1 \cap X_{s1} = 0)\big| X_{s0} \neq 2\big)$$

$$= \mathrm{P}(X_{s12} = 2 | X_{s1} = 1, X_{s0} \neq 2) \times \mathrm{P}(X_{s1} = 1 | X_{s0} \neq 2)$$

$$+\mathrm{P}(X_{s12} = 2 | X_{s1} = 0, X_{s0} \neq 2) \times \mathrm{P}(X_{s1} = 0 | X_{s0} \neq 2)$$

$$+\mathrm{P}(X_{s2} = 1 | X_{s1} = 1, X_{s0} \neq 2) \times \mathrm{P}(X_{s1} = 1 | X_{s0} \neq 2)$$

$$+\mathrm{P}(X_{s2} = 1 | X_{s1} = 0, X_{s0} \neq 2) \times \mathrm{P}(X_{s1} = 0 | X_{s0} \neq 2)$$

$$= \mathrm{P}(X_{s12} = 2 | X_{s1} = 1) \times \mathrm{P}(X_{s1} = 1 | X_{s0} \neq 2)$$

$+\mathrm{P}(X_{s12} = 2|X_{s1} = 0) \times \mathrm{P}(X_{s1} = 0|X_{s0} \neq 2)$

$+\mathrm{P}(X_{s2} = 1|X_{s1} = 1) \times \mathrm{P}(X_{s1} = 1|X_{s0} \neq 2)$

$+\mathrm{P}(X_{s2} = 1|X_{s1} = 0) \times \mathrm{P}(X_{s1} = 0|X_{s0} \neq 2)$ (2)

Let $P_{ij}(t)$ denote the transition probability from state *i* to state *j* in a time interval *t*, where *i*, *j*=0, 1, and 2 and $\sum_{j=0}^{2} P_{ij}(t) = 1$ for any *i*. Note that $P_{ij}(t) = 0$ for *i*>*j* because the spontaneous regression of PrCa is not allowed. The first part of equation (2) is the transition probability from PCDP at the first screen to the CP between the first and second screen during the inter-screening interval (in this trial, 4 years) times being in the PCDP at the first screen at aged *A* conditional on not developing clinical symptoms before the first screen. The former can be expressed as $P_{12}(4)$ and the latter as $\frac{P_{01}(A)}{P_{00}(A)+P_{01}(A)}$.

Therefor, equation (2) can be written as

$$P_{12}(4) \times \frac{P_{01}(A)}{P_{00}(A) + P_{01}(A)} + P_{02}(4) \times \frac{P_{00}(A)}{P_{00}(A) + P_{01}(A)}$$

$$+P_{11}(4) \times \frac{P_{01}(A)}{P_{00}(A) + P_{01}(A)} + P_{01}(4) \times \frac{P_{00}(A)}{P_{00}(A) + P_{01}(A)}$$

$$= (P_{12}(4) + P_{11}(4)) \times \frac{P_{01}(A)}{P_{00}(A) + P_{01}(A)} + (P_{02}(4) + P_{01}(4)) \times \frac{P_{00}(A)}{P_{00}(A) + P_{01}(A)}$$

Given the constraint of $\sum_{j=0}^{2} P_{ij}(t) = 1$, we have

$P_{12}(4) + P_{11}(4) = 1$ and $P_{02}(4) + P_{01}(4) = 1 - P_{00}(4)$

The formula could be furthered modified as follows

$$= \frac{P_{01}(A)}{P_{00}(A) + P_{01}(A)} + (1 - P_{00}(4)) \times \frac{P_{00}(A)}{P_{00}(A) + P_{01}(A)}$$

$$= \frac{P_{01}(A)}{P_{00}(A) + P_{01}(A)} + \frac{P_{00}(A)}{P_{00}(A) + P_{01}(A)} - P_{00}(4) \times \frac{P_{00}(A)}{P_{00}(A) + P_{01}(A)}$$

$$= \frac{P_{01}(A) + P_{00}(A)}{P_{00}(A) + P_{01}(A)} - P_{00}(4) \times \frac{P_{00}(A)}{P_{00}(A) + P_{01}(A)}$$

$$= 1 - P_{00}(4) \times \frac{P_{00}(A)}{P_{00}(A)+P_{01}(A)} \qquad (2)$$

To derive the transition probability (P(t)), we use the Kolmogorov differential equations which has the same unique solution with forward equations

$$\boldsymbol{P'(t) = P(t)Q}$$

and backward equations (Cox, & Miller, 1965)

$$\boldsymbol{P'(t) = QP(t),}$$

where **Q** is the intensity matrix of transition rates can be expressed as

$$Q = \begin{bmatrix} -\lambda_1 & \lambda_1 & 0 \\ 0 & -\lambda_2 & \lambda_2 \\ 0 & 0 & 0 \end{bmatrix}$$

If the matrix **Q** is finite with initial condition $\boldsymbol{P(0) = I}$, the formal solution of transition probability matrix can be derived as

$$\boldsymbol{P(t)} = exp(\boldsymbol{Q}t)$$

Therefore, the formula (2) could be represented as

$$1 - (e^{-\lambda_1 \times 4}) \times \frac{e^{-\lambda_1 \times A}}{e^{-\lambda_1 \times A} + \frac{\lambda_1 (e^{-\lambda_2 \times A} - e^{-\lambda_1 \times A})}{(\lambda_1 - \lambda_2)}} \qquad (3)$$

The probability of surfacing to clinical phase as interval cancer arising from the test negative cases

The probability of surfacing to the clinical phase like interval cancers (IC) during the four years of inter-screening interval, say $P_{IC}(4)$, for subjects with PSA less than 4ng/ml would be used to approximate the parameters of transition rate.

The general formula for inter-screening interval with t is

$$P_{IC}(t) = \int_0^t \lambda_1 e^{-\lambda_1 s} \int_0^{t-s} \lambda_2 e^{-\lambda_2 u}\, duds = \frac{\lambda_1(1-e^{-\lambda_2 \times t}) - \lambda_2(1-e^{-\lambda_1 \times t})}{(\lambda_1-\lambda_2)} \quad (4)$$

where **s** is the time at which the tumour enter the PCDP and **u** is the time at which it develops form the PCDP to CP ($P_{12}(t)$).

According to formula (4)

$$P_{IC}(t) = \frac{\lambda_1(1-e^{-\lambda_2 t}) - \lambda_2(1-e^{-\lambda_1 t})}{(\lambda_1-\lambda_2)} = \frac{\lambda_1 - \lambda_1 e^{-\lambda_2 t} - \lambda_2 + \lambda_2 e^{-\lambda_1 t}}{(\lambda_1-\lambda_2)}$$

Using the Maclaurin's series and omitting the extremely small components, we have

$$e^{-\lambda_1 t} = 1 + \frac{(-\lambda_1 t)^1}{1!} + \frac{(-\lambda_1 t)^2}{2!} + \frac{(-\lambda_1 t)^3}{3!} + \cdots = 1 - \lambda_1 t \quad \text{if } \lambda_1 \to 0$$

So the formula could be simplified as

$$P_{IC}(t) = \frac{\lambda_1 - \lambda_1 e^{-\lambda_2 t} - \lambda_2 + \lambda_2 e^{-\lambda_1 t}}{(\lambda_1-\lambda_2)} = \frac{\lambda_1 - \lambda_1 e^{-\lambda_2 t} - \lambda_1\lambda_2 t}{(\lambda_1-\lambda_2)}$$

After some algebra by multiplying $(\lambda_1 - \lambda_2)$ at both side of the formula, we have

$$\lambda_1 = \frac{\lambda_2 P_{IC}(t)}{P_{IC}(t) - 1 + e^{-\lambda_2 t} + \lambda_2 t} \quad (5)$$

We assumed the λ_2, the inverse of mean sojourn time (MST), would be the same regardless of PSA level at baseline once the tumour enters the PCDP. MST of 6.75

years according to above-mentioned analysis was used. Let t=4 according to our screen trial design and we could get the observed value of interval cancer during 4 years inter-screen interval, $P_{IC}(t)$. λ_1 could be estimated.

Based on the assumption that λ_1 is affected by PSA level, six parameters of λ_1 corresponding to 6 PSA level subgroups would be estimated by using the formula (5) mentioned above.

Based on the formulas above, λ_1 was estimated and was used to calculate the expected number of asymptomatic prostate cancer among subjects with the PSA level below 4 ng/ml by using the following formula

$$P_{PCDP} = P(X(\text{at } 1^{\text{st}} \text{ screen}) = 1 | X(\text{before } 1^{\text{st}} \text{ screen}) \neq 2) \quad (6)$$

The following formula gives the probability of staying in PCDP of given specific age.

$$P_{PCDP}(\text{A}) = \frac{P_{01}(A)}{P_{00}(A)+P_{01}(A)} = \frac{\frac{\lambda_1(e^{-\lambda_2\times A}-e^{-\lambda_1\times A})}{(\lambda_1-\lambda_2)}}{e^{-\lambda_1\times A}+\frac{\lambda_1(e^{-\lambda_2\times A}-e^{-\lambda_1\times A})}{(\lambda_1-\lambda_2)}} \quad (7)$$

The expected number of asymptomatic prostate cancer for the screenees with the PSA level below 4ng/ml, saying $N_{PCDP}^{PSA_1<4}$, in *i* PSA level can be computed as follows.

$$N_{PCDP\ i}^{PSA_1<4} = N_{i}^{PSA_1<4} \times P_{PCDP\ i}^{PSA_1<4} \quad (8)$$

Reference

1. Duffy SW, Chen HH, Tabar L, Day NE. Estimation of mean sojourn time in breast cancer screening using a Markov chain model of both entry to and exit from the preclinical detectable phase. Stat Med. 1995;14:1531-1543.

Supplementary Table 1: Sensitivity analysis of the estimated pre-clinical detectable phase cancers by PSA level with six different mean sojourn time for population-based PSA screening

PSA (ng/ml)	Number	Observed ICs	Incidence of PCDP (λ_1)	P_{PCDP}	Estimated PCDP Cases
Given MST=5					
0.00~0.99	9614	5	0.000416	0.0021	20
1.00~1.99	6112	17	0.002207	0.0110	67
2.00~2.49	1342	7	0.004098	0.0205	27
2.50~2.99	822	11	0.010188	0.0509	42
3.00~3.49	623	13	0.015446	0.0772	48
3.50~3.99	441	9	0.015132	0.0757	33
Total	18954	62	0.002590	0.0129	245
Given MST=6.5					
0.00~0.99	9614	5	0.000512	0.0033	32
1.00~1.99	6112	17	0.002698	0.0175	107
2.00~2.49	1342	7	0.004983	0.0324	43
2.50~2.99	822	11	0.012168	0.0791	65
3.00~3.49	623	13	0.018170	0.1181	74
3.50~3.99	441	9	0.017816	0.1158	51
Total	18954	62	0.003163	0.0206	390
Given MST=6.75					
0.00~0.99	9614	5	0.000528	0.0036	34
1.00~1.99	6112	17	0.002779	0.0188	115
2.00~2.49	1342	7	0.005128	0.0346	46
2.50~2.99	822	11	0.012479	0.0842	69
3.00~3.49	623	13	0.018583	0.1254	78
3.50~3.99	441	9	0.018225	0.1230	54
Total	18954	62	0.003258	0.0220	417
Given MST=7					
0.00~0.99	9614	5	0.000544	0.0038	37
1.00~1.99	6112	17	0.002860	0.0200	122
2.00~2.49	1342	7	0.005271	0.0369	50
2.50~2.99	822	11	0.012785	0.0895	74
3.00~3.49	623	13	0.018985	0.1328	83

3.50~3.99	441	9	0.018623	0.1303	57
Total	18954	62	0.003352	0.0235	445
Given MST=10					
0.00~0.99	9614	5	0.000734	0.0073	70
1.00~1.99	6112	17	0.003805	0.0379	232
2.00~2.49	1342	7	0.006905	0.0688	92
2.50~2.99	822	11	0.015988	0.1590	131
3.00~3.49	623	13	0.022884	0.2271	141
3.50~3.99	441	9	0.022494	0.2233	98
Total	18954	62	0.004445	0.0443	840

ICs: Interval cancers

P_{PCDP}: Probability of cancer stayed at detectable phase

MST: Mean sojourn time

Supplementary Figure 1: Sensitivity analysis of the corrected ROC curves with six different mean sojourn time for population-based PSA screening

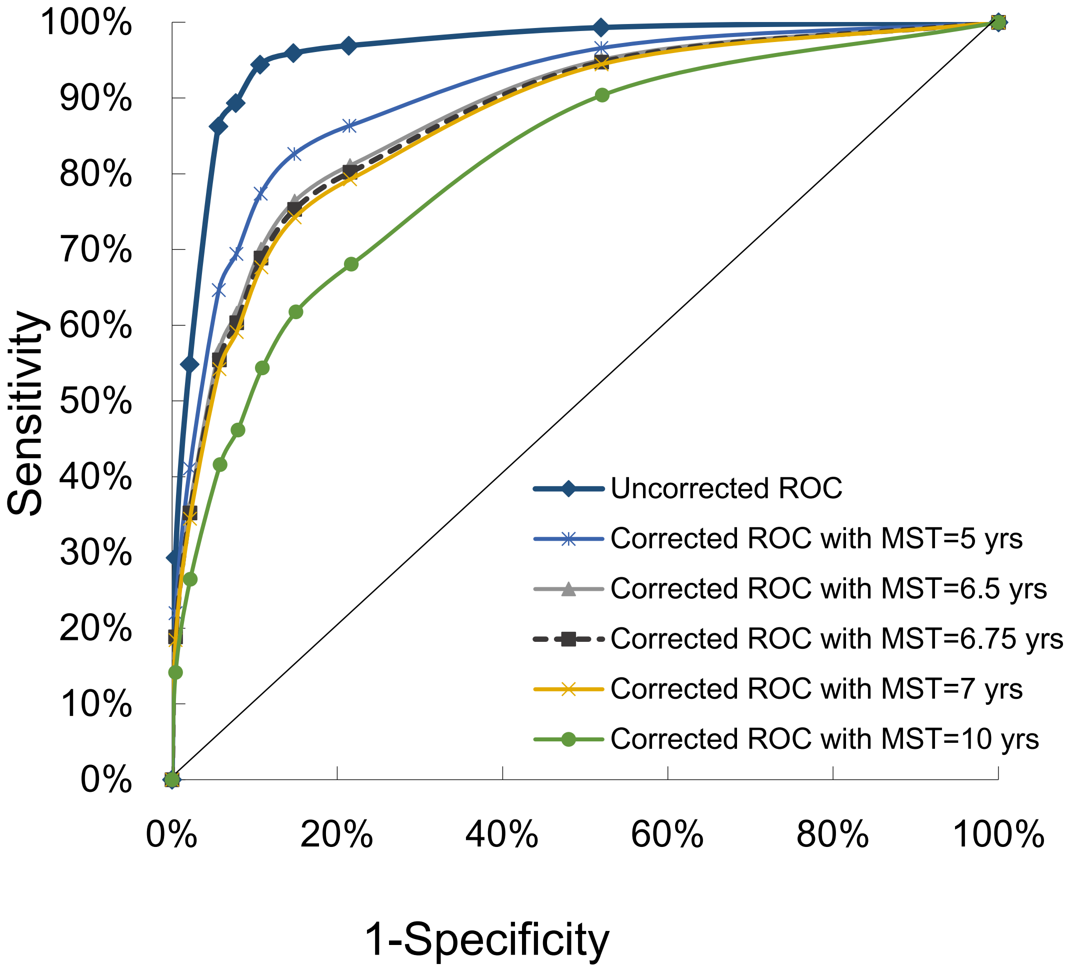

MST	AUC (95% CI)	Difference in AUC (95% CI)
Uncorrected	95.9% (95.3%-96.6%)	-
5	80.9% (78.9%-83.0%)	15.0% (12.9%-17.2%)
6.5	77.6% (75.5%-79.7%)	18.4% (16.2%-20.6%)
6.75	77.0% (74.9%-79.1%)	18.9% (16.7%-21.1%)
7	76.5% (74.4%-78.6%)	19.4% (17.2%-21.7%)
10	71.0% (68.9%-73.2%)	24.9% (22.7%-27.2%)
